# Effects of Jaeumkanghwa-tang on tamoxifen responsiveness in preclinical ER+ breast cancer model

**DOI:** 10.1530/ERC-18-0393

**Published:** 2019-01-14

**Authors:** Fabia De Oliveira Andrade, Wei Yu, Xiyuan Zhang, Elissa Carney, Rong Hu, Robert Clarke, Kevin FitzGerald, Leena Hilakivi-Clarke

**Affiliations:** Department of Oncology, Georgetown University, Washington, District of Columbia, USA

**Keywords:** Jaeumkanghwa-tang, tamoxifen, breast cancer, endometrial atypical hyperplasia, preclinical

## Abstract

Resistance to endocrine therapy remains a clinical challenge in the treatment of estrogen receptor-positive (ER+) breast cancer. We investigated if adding a traditional Asian herbal mixture consisting of 12 herbs, called Jaeumkanghwa-tang (JEKHT), to tamoxifen (TAM) therapy might prevent resistance and recurrence in the ER+ breast cancer model of 7,12-dimethylbenz[a]anthracene (DMBA)-exposed Sprague–Dawley rats. Rats were divided into four groups treated as follows: 15 mg/kg TAM administered via diet as TAM citrate (TAM only); 500 mg/kg JEKHT administered via drinking water (JEKHT only group); TAM + JEKHT and no treatment control group. The study was replicated using two different batches of JEKHT. In both studies, a significantly higher proportion of ER+ mammary tumors responded to TAM if animals also were treated with JEKHT (experiment 1: 47% vs 65%, *P* = 0.015; experiment 2: 43% vs 77%, *P* < 0.001). The risk of local recurrence also was reduced (31% vs 12%, *P* = 0.002). JEKHT alone was mostly ineffective. In addition, JEKHT prevented the development of premalignant endometrial lesions in TAM-treated rats (20% in TAM only vs 0% in TAM + JEKHT). Co-treatment of antiestrogen-resistant LCC9 human breast cancer cells with 1.6 mg/mL JEKHT reversed their TAM resistance in dose–response studies *in vitro*. Several traditional herbal medicine preparations can exhibit anti-inflammatory properties and may increase anti-tumor immune activities in the tumor microenvironment. In the tumors of rats treated with both JEKHT and TAM, expression of *Il-6* (*P* = 0.03), *Foxp3*/T regulatory cell (Treg) marker (*P* = 0.033) and *Tgfβ1* that activates Tregs (*P* < 0.001) were significantly downregulated compared with TAM only group. These findings indicate that JEKHT may prevent TAM-induced evasion of tumor immune responses.

## Introduction

Medicine practiced in Asian countries can be a mixture of traditional Asian and modern Western medicine. Traditional medicine practitioners use herbal preparations (Zhang* et al.* 2015, Zhou* et al.* 2016) and various mind and body practices, including acupuncture and tai chi (Tao* et al.* 2016), while Western medicine relies heavily on the use of pharmaceuticals. Herbs are commonly taken by Asian cancer patients to reduce the side effects of Western treatments, such as radiation and chemotherapy, and to improve well-being (Fouladbakhsh* et al.* 2013). In a cross-sectional survey done among 1498 cancer patients in the United Kingdom, 22.7% of predominantly white breast cancer patients reported using herbal supplements (Damery* et al.* 2011). Thus, also among Western patients herbal preparations are used.

Herbs have been proposed to increase sensitivity to cancer treatments and prevent recurrence and metastasis (Xiu* et al.* 2015). However, compelling experimental evidence to support the efficacy of herbs in cancer patients, and the identification of the biological pathways involved in mediating their effects, are often absent from the literature. *In vitro* studies show that various herbs can inhibit the growth of cancer cells (Bonofiglio* et al.* 2016) – observations that are sometimes replicated *in vivo* in mice (Chen* et al.* 2016). However, these studies often use doses of herbs that are not pharmacologically relevant for humans and the results cannot easily be extrapolated to predict clinical benefit. Studies done *in vitro* or *in vivo* in immunocompromised mice also cannot address the role of an intact immune system. Nonetheless, many herbs may suppress inflammation and affect the immune system (Ghasemian* et al.* 2016, Yatoo* et al.* 2018), activities that play a critical role in cancer development (Finn 2018). It remains largely unknown if herbal preparations provide any significant survival benefit for cancer patients.

Endocrine therapy is widely used in the treatment of ER+ breast cancer, reflecting its effectiveness in both adjuvant and metastatic disease (Smith 2014, Ziauddin* et al.* 2014). ER+ breast cancers comprise approximately 70% of all breast cancers (Lim* et al.* 2012, DeSantis* et al.* 2017). The most commonly used endocrine therapy agents are selective estrogen receptor modulators (SERMs) such as tamoxifen (TAM) for premenopausal patients and aromatase inhibitors like letrozole for postmenopausal patients (Baumann & Castiglione-Gertsch 2009, Komm & Mirkin 2014). Unfortunately, resistance to endocrine therapies and consequent disease recurrence poses a major obstacle in the successful treatment of ER+ breast cancers. Recurrence often reflects transition to a more aggressive phenotype that is very difficult to eradicate. The clinical reality is that up to 52% of ER+ breast cancer patients with localized disease recur during or after endocrine therapy in patients that are followed for up to 20 years after diagnosis (Pan* et al.* 2017). TAM also has moderate (menopausal-like symptoms) to severe side effects (an increased risk of developing endometrial cancer) (Bergman* et al.* 2000, Jones* et al.* 2012) and compliance is variable with many patients not completing their treatment regimen (Murphy* et al.* 2012, Chirgwin* et al.* 2016). Studying the factors that cause the development of endocrine resistance is one of the top priorities in breast cancer research (Clarke* et al.* 2011), as is identifying ways to reduce menopausal symptoms, joint pain (arthralgia), thromboembolic events and the risk of endometrial cancer to increase compliance with treatment.

We studied here whether intake of a 12 herb mixture called Jaeumkanghwa-tang (JEKHT) (Jung *et al.* 2010) (Table 1) modifies the response of ER+ mammary tumors in Sprague–Dawley rats to TAM. We have previously used the carcinogen-based ER+ mammary cancer model, a well-characterized model (Russo* et al.* 1990) used originally by Dr V.C. Jordan to establish TAM as an endocrine therapy (Jordan 1997), to study factors programming for endocrine resistance (Hilakivi-Clarke* et al.* 2016, Zhang* et al.* 2017). In addition, we explored here if JEKHT affects development of the premalignant endometrial changes linked to TAM use, as has been reported for other herbal mixtures (Burke* et al.* 1996, Tsai* et al.* 2014, Hu* et al.* 2015). JEKHT is a traditional herbal medicine used in Korea, China and Japan for various purposes, especially to treat normal age-related pathophysiology, such as impaired hearing and vision and lack of energy. It also is used to treat gynecological health problems (Sekiya *et al.* 2003) or allergic inflammatory reactions (Kim* et al.* 2004). In studies performed *in vitro* and *in vivo* in immunocompromised mice, JEKHT inhibited the growth of several cancer cell lines (Kim* et al.* 2015). In immunocompetent rats, JEKHT inhibited the development of benign prostatic hyperplasia (Shin* et al.* 2012). One report noted that JEKHT is used by breast cancer patients to relieve hot flashes caused by an endocrine therapy (Zheng *et al*. 2010). Among the potential mechanisms of action of JEKHT are suppression of NFkB and inflammatory cytokines (Kim *et al.* 2004) and stimulation of the immune system (Jung *et al.* 2010).

**Table 1:** JEKHT composition.

Herb	Common name	Content in experiment 1 (g/100 g)	Content in experiment 2 (g/100 g)
*Paeoniae Radix*	Paeonia	8.75	10.20
*Angelicae Gigantis Radix*	Korean angelica root	11.10	10.20
*Asparagi Tuber*	Asparagus cochinchinensis Merr	12.51	10.20
*Atractylodis Rhizoma Alba*	White atractylis	9.22	12.29
*Rehmanniae Radix Crudus*	Rehmannia glutinosa	16.27	10.20
*Citri Unshii Pericarpium*	Dried orange peel	5.64	10.20
*Anemarrhenae Rhizoma*	Anemarrhena	4.70	6.14
*Phellodendri Cortex*	Phellodendron bark	2.73	6.14
*Glycyrrhizae Radix et Rhizoma*	Licorice	3.39	6.14
*Zingiberis Rhizoma Crudus*	Ginger	2.45	4.05
*Liriopis Tuber*	Lilyturf	7.53	10.20
*Zizyphi Fructus*	Jujube	15.71	4.05

We found that JEKHT increased the response of ER+ mammary tumors to TAM and reduced tumor recurrence. In addition, JEKHT inhibited the formation of premalignant endometrial lesions. These effects were associated with a significant downregulation of cytokine *Il-6*, and tumor immunosuppressive markers *Foxp3* and *Tgfβ1* in mammary tumors of rats treated with TAM + JEKHT.

## Methods

### Animals and breeding

Sprague–Dawley rats (Harlan, USA) were used in all experiments. Animals were housed in a temperature and humidity controlled room under a 12-h light-darkness cycle and fed AIN93G laboratory diet obtained from Harlan Laboratories (Madison, WI, USA) throughout the study. All animal procedures were approved by the Georgetown University Animal Care and Use Committee, and the experiments were performed following the National Institutes of Health guidelines for the proper and humane use of animals in biomedical research.

### Mammary tumorigenesis

Mammary tumors were induced by the administration of 10 mg of 7,12-dimethylbenz[a]anthracene (DMBA) (Sigma) in 1 mL of peanut oil by oral gavage to 50-day-old rats. Animals were examined for mammary tumors by palpation once per week, starting 3 weeks post DMBA treatment. Tumor growth was measured using a caliper, and the length and width of each tumor were recorded. During the tumor monitoring period, animals in which tumor burden approximated 10% of total body weight were killed, as required by our institution’s ethical guidelines.

### Tamoxifen and JEKHT treatments

Two separate experiments were done using two different batches of JEKHT. The first experiment had three treatment arms: (1) 337 ppm tamoxifen citrate (TAM, obtained from Sigma and administered via AIN93G diet, resulting in approximately 15 mg/kg body weight daily tamoxifen dose; *n* = 12 animals that by the end of tumor monitoring period had developed 29 tumors), (2) 500 mg/kg body weight JEKHT per day (administered via drinking water; *n* = 8 animals with 43 tumors) and (3) TAM + JEKHT combination (*n* = 17 animals with 32 tumors). Additional controls were nine DMBA-treated rats with 37 tumors that did not receive any treatment. Treatments were started when a rat had developed their first mammary tumors that measured 11–13 mm in diameter. The second experiment consisted of only two groups: those treated with 340 ppm TAM (*n* = 19 rats with 58 tumors) and those treated with 500 mg/kg body weight JEKHT + 340 ppm TAM (*n* = 17 rats with 44 tumors). In this study, treatments started when the first tumor in a rat reached 11 mm in diameter.

JEKHT was produced by Hanjung Pharmaceuticals (165-7 Sangseo-dong, Daedeok-gu, Daejeon, Korea) based on the formulation approved by Korean Ministry of Food and Drug Safety (MFDS). This company manufactures JEKHT under the Good Manufacturing Practice (GMP) guideline by MFDS. All individual herbs are within the specification of Korean Pharmacocopia 11th edition, and the final quality control is performed by analysis of three index materials: berberine, glycyrrhizic acid and paeoniflorin. In our study, JEKHT was in a form of a powder and was used for the study before expiration date. The lot number in experiment 1 was KKG3003, and MJK701 in experiment 2. The composition of both lots of JEKHT is shown in Table 1.

### Response of tumors to treatments

Based on their response to TAM, tumors were divided into four categories: those exhibiting (1) *response* (tumor disappeared), (2) *partial response* (PR, tumor stopped growing and/or started to shrink), (3) *de novo* resistance (tumor continued growing, including tumors that were not present at the start of the treatment) and (4) *acquired resistance* (tumors that exhibited response lasting >4 weeks and then recurred at the same location where they were initially observed). Macroscopic confirmation of recurrence was performed at autopsy based on the location of the primary tumor. Further, when a tumor recurred, the tumor had to reach a size of at least 1.3 cm in diameter (criteria of starting TAM treatment) to be assigned as exhibiting acquired resistance/recurrence. Monitoring responses continued up to 20 weeks after starting treatments.

### Tissue collection

At the end of the tumor monitoring period, rats were killed by CO_2_. At necropsy, blood was collected by cardiac puncture, and mammary glands, tumors and endometrial tissues were removed and either flash frozen in liquid N_2_ for future analysis or fixed in 10% formalin for histopathology purposes.

### Tumor pathologic evaluation

Formalin-fixed mammary tumors and endometrial tissues were embedded in paraffin and cut into 5 µm sections. Hematoxylin and eosin (H&E)-stained sections were then used for histopathological evaluation that was done by a veterinary pathologist, either at ARUP laboratories (Salt Lake City, Utah) in experiment 1 or by Dr Galli, a newly appointed veterinary pathologist in our institution (experiment 2).

### Protein isolation and immunoblotting

Protein isolated from malignant mammary tumors was used to determine whether the three treatment arms differed from each other regarding the expression of estrogen receptor alpha (ERα), ERβ and progesterone receptor (PgR) by Western blot. Briefly, 100 mg of frozen mammary tumor or endometrial tissue was ground using a mortar and pestle in liquid nitrogen and lysed in RIPA lysis buffer (1% NP-40, 0.1% SDS, 50 mM Tris–HCl pH 7.4, 150 mM NaCl, 0.5% sodium deoxycholate, 1 mM EDTA, 1 mM sodium orthovanadate, 10 mM glycerophosphate, 5 mM pyrophosphate and 1 mM PMSF) with Complete Mini Protease Inhibitor (Roche). Cell debris and chromatin were precipitated by centrifugation and discarded. Protein concentration was measured using the BCA Protein Assay Kit (Thermo Scientific) according to the manufacturer’s protocol. Thirty micrograms of the protein were separated on a NuPage 4–12% Bis-Tris gel (Life Technologies) and transferred to nitrocellulose via iBlot Transfer Stack and Blotting System (Life Technologies). The nitrocellulose membrane was blocked in 5% bovine serum albumin (BSA) in TBS with 0.1% Tween-20 (0.1% TBST) at room temperature for 1 h and incubated with antibodies (diluted in TBST) against the following: ERα (VP-E613, Vector Laboratories), PgR (ab90577, Abcam) and ERβ (E1276, Sigma). Membranes were incubated with primary antibodies at 4°C with gentle shaking overnight, followed by washing three times with TBST for 10 min each. The membrane was incubated with horseradish peroxidase (HRP)-conjugated secondary antibody (1:5000 dilution, Santa Cruz) in TBST plus 1% milk at room temperature for 1 h, followed by three 10 min washes in TBST. HyGLO Chemiluminescent HRP antibody detection spray was applied to the membrane and the signal was detected in autoradiography films in a dark room. The protein level was determined by the intensity of the bands using the Quantity One software (Bio-Rad).

### Immunohistochemistry (IHC)

To assess CD8+ infiltration in mammary tumors, IHC assays were performed. Five micrometer paraffin sections, cut transversely, were deparaffinized and rehydrated from xylene through a graded series of ethanol. Antigen retrieval was performed in a high-pH Target Retrieval Solution (pH 9, DAKO S2368) in a microwave for 15 min, followed by 20 min of cooling at room temperature. Then, endogenous peroxidases were blocked with 3% hydrogen peroxide in water (DAKO, K0679) and non-specific staining was blocked with protein block serum-free (DAKO, X0909). Immunostaining with CD8 (Abcam, ab33786, 1:100) was performed overnight following the manufacturer’s protocol for LSAB-HRP immunohistochemistry kit (DAKO, K0679). Slides were examined under a bright-field microscope (Olympus BX 61 with Qiacam scanning camera) and image software package CellSens (Waltham). Micrographs were taken at 20× magnification. Total number of positive cells per field was calculated.

### mRNA levels

RT-qPCR was used to determine differences in *Foxp3* and *Tgfβ1* mRNA expression in the mammary tumors among the three treatment arms.

#### RNA extraction and cDNA synthesis

Fifty micrograms of frozen mammary tumor was ground using a mortar and pestle in liquid nitrogen. Total RNA was extracted using TRIzol reagent (Life technologies) followed by one step of DNase I treatment to prevent genomic DNA contamination (Roche), as the manufacturer instructed. Quantity and quality of RNA was determined according to the optical density ratio (OD260:OD280) using a ND1000 NanoDrop Spectrophotometer (Thermo Scientific). A total of 2 µg RNA per sample were used to generate cDNA via reverse transcription in a PTC-100 thermal cycler (Bio-Rad) using the following steps: initiation at 25°C for 10 min, reverse transcribing at 37°C for 2 h and deactivation at 85°C for 5 min.

#### Quantitative real-time PCR

Briefly, 12.5 μg cDNA was used as template with primers specific for Foxp3 and Hprt using 5 µL Absolute QPCR SYBR Green ROX Mix in a 10 µL reaction (Thermo Scientific). Primer sequences are shown in Supplementary Table 1 (see section on supplementary data given at the end of this article). Serially diluted cDNA samples (100 to 0.032 ng/µL) were included with each primer. To determine the relative quantity of the gene, the expression was normalized to the level of the house-keeping gene Hprt. Real-time PCRs were carried out in an ABI Prism 7900 Sequence Detection System (Life Technologies) with the following thermo cycler setting: activation of the enzyme at 95°C for 15 min, 40 cycles of denaturing at 95°C for 15 s, annealing at 60°C for 30 s and elongation at 72°C for 30 s, followed by one step of dissociation to ensure the purity of the product. Primers used in the real-time PCR were designed using Vector NTI software (Life Technologies). The result of the reaction was checked and exported using SDS 2.3 software (Life Technologies). The highest efficiency of the machine was confirmed by ensuring that the *R* ^2^ of the standard curve was >0.98 and that the slope was within 3.3 ± 0.3.

### Cytokine levels

The levels of interleukin-6 (IL-6) and interferon gamma (IFN-γ) were determined in the circulation, and tissue levels of IL-1b, IL-10 and TNF-α were determined in the spleen of animals treated with TAM, JEKHT or their combination. We also determined mRNA expression of *Il-6*, *Il-10* and *Ifn-γ* in mammary tumors, using methods described earlier. Primer sequences for these genes are shown in Supplementary Table 1. Samples were obtained from the animals at the end of the tumor monitoring period.

IL-6 and IFN-γ concentrations in serum were measured by enzyme-linked immunosorbent assay (ELISA) kits (IL-6: R&D Systems Inc., and IFN-γ: BD Biosciences/Pharmingen) as pg/mL following manufacturer’s recommended protocols. Splenic cytokine content measurements were done using the following ELISA kits: TNF-α from BD Biosciences/Pharmingen and IL-1β and IL-10 from Genzyme. Approximately 10–15 mg of tissue samples were homogenized in a tissue grinder containing 1 mL of lysis buffer (PBS with 2 mM PMSF and 1 mg/mL of aprotinin, leupeptin and pepstatin A). Analysis was performed with 100 mL of lysis buffer, containing 0, 10, 50 or 100 mL of tissue homogenate. Each sample was run in duplicate. A standard curve was generated for each assay, based on replicates of the measured absorbance. The average coefficient of variance was less than 10%.

### XTT cell proliferation assay

LCC9 cells were derived from ER+ MCF-7 human breast cancer cells and selected for resistance to both TAM and fulvestrant (lCl 182,780) *in vitro* and by serial xenografting in ovariectomized nude mice (Brunner* et al.* 1993, 1997). LCC9 cells remained ER+. Five thousand LCC9 breast cancer cells were seeded in 96-well plates. After 16 h, 1.6 mg/mL of JEKHT was added to LCC9 cells in the presence of increasing doses of 4-hydroxy-tamoxifen (4-OHT). Equal volume of DMSO was added to cells as negative control. Six days post drug treatment, XTT assay (Sigma) was performed to measure cell proliferation. Briefly, cells were washed with PBS and incubated with fresh serum-free media containing 1 mg/mL XTT and 6 µg/mL phenazine methosulfate (Sigma) for 4 h, followed by microplate reading at absorption of 450 nm.

### Statistical analysis

Body and organ weights, serum cytokine levels, tumor multiplicity and receptor mRNA levels were determined using one-way ANOVA, and the difference was considered significant if the *P* value was less than 0.05. Fisher’s least significant difference (LSD) test was applied as a *post hoc* analysis when the one-way ANOVA result showed significance among groups. Data for cytokine mRNA levels in benign and malignant tumors, and response to TAM or TAM + JEKHT in LCC9 breast cancer cells were analyzed using two-way ANOVA. CD8a-positive T cells and mRNA expression of *Foxp3* and *Tgfβ1* in partially responding and resistant tumors among TAM only, JEKHT only or TAM+JEKHT were analyzed using three-way ANOVA. *Post hoc* analysis was done using the Holm–Sidak method. Chi^2^ analysis was applied to determine the statistical significance in response of tumors to TAM vs TAM + JEKHT or to JEKHT vs no treatment.

## Results

### Effects on TAM responsiveness

#### JEKHT reduces *de novo* and acquired resistance to TAM

**Figure 1:**
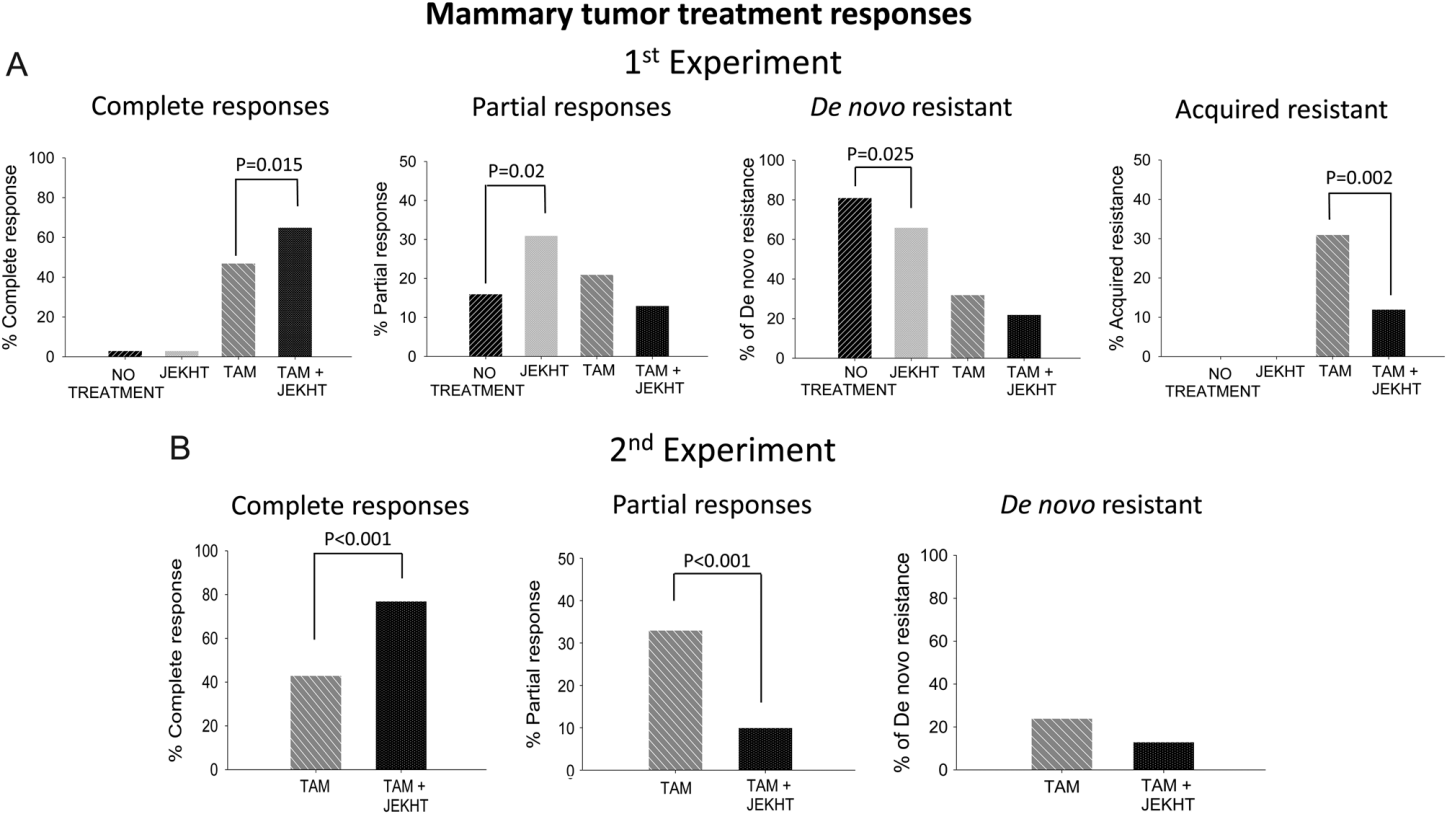
Responses of mammary tumors to tamoxifen (TAM) therapy. Percentage of complete responses, PRs, *de novo* resistance and acquired resistance in rats that were treated with TAM or TAM + JEKHT. In JEKHT only treated rats or in rats not receiving any treatment, percentage of tumors that disappeared, did not grow or grew also are shown. (A) In experiment 1, numbers of tumors were 29 in TAM, 32 in TAM + JEKHT, 43 in JEKHT only and 37 in no treatment group. TAM + JEKHT increased complete responses (*P* = 0.015) and reduced tumors exhibiting acquired resistance (*P* = 0.002). JEKHT only treated rats exhibited more non-growing tumors than rats that did not receive any treatment (*P* = 0.02) and less growing tumors (*P* = 0.025). (B) In experiment 2, TAM group consisted of 58 tumors and TAM+JEKHT group of 44 tumors. Adding JEKHT significantly increased complete responses (*P* < 0.001).

When mammary tumors reached a size of 13 mm in diameter, rats were given 337 ppm TAM via food, resulting in a daily intake of 15 mg/kg body weight. In experiment 1, among TAM only treated rats, 47% of all tumors responded and disappeared (complete response), 21% exhibited a PR and stopped growing; 32% were resistant (continued growing) (Fig. 1A). Adding 500 mg/kg body weight JEKHT via drinking water to the treatment regimen increased the response rate to 65% and reduced the *de novo* resistance rate to 22%. The increase in completely responding tumors in the TAM + JEKHT group was statistically significant (*P* = 0.015). In both the TAM and TAM + JEKHT groups, some completely responding tumors recurred (Fig. 1A). The rate of recurrence was 31% in the TAM only treated rats but only 12% in the TAM + JEKHT-treated rats (*P* = 0.002).

In experiment 2, TAM + JEKHT also significantly increased the rate of complete responses (77%), compared with TAM only treated rats (43%, *P* < 0.001) (Fig. 1B). Recurrences could not be assessed in experiment 2, as all rats were removed from this study 9 weeks after exhibiting a complete response.

JEKHT alone had a small, but significant effect on tumor growth when compared with tumors in rats not receiving any treatment; 66 vs 81% were resistant or grew (*P* = 0.025), 31 vs 16% exhibited a PR (*P* = 0.02) and 3 vs 3% disappeared after being initially detected (Fig. 1A).

We also determined if JEKHT might prevent the development of new tumors during TAM treatment. At the end of tumor monitoring period, respectively, 32 and 28% in experiments 1 and 2 of all tumors appeared during TAM treatment; 26 and 32% appeared during TAM + JEKHT treatment. Thus, while JEKHT improved the initial response to TAM and prevented recurrence of the responding tumors, it did not prevent the development of new tumors compared with TAM only treatment. In experiment 1, both TAM (mean ± s.e.m.; 2.4 ± 0.4, *P* = 0.004) and TAM + JEKHT (1.9 ± 0.3, *P* < 0.001)-treated rats had a significantly lower mammary tumor multiplicity than rats treated with JEKHT only (5.42.4 ± 0.5). TAM + JEKHT-treated rats, but not TAM only treated rats, also had significantly lower tumor multiplicity than rats not treated with anything (4.12.4 ± 1.0, *P* = 0.02). In experiment 2, tumor multiplicity in TAM only treated group was 3.1 ± 0.5 and 2.6 ± 0.4 in TAM + JEKHT-treated group.

#### JEKHT sensitizes antiestrogen-resistant human LCC9 breast cancer cells to TAM

**Figure 2:**
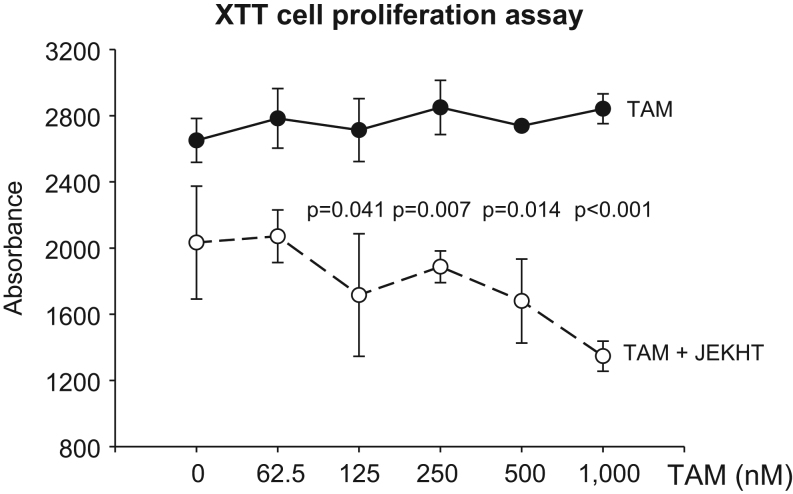
Response of antiestrogen resistant LCC9 human breast cancer cells to JEKHT. Response of antiestrogen resistant LCC9 human breast cancer cells to 4-hydroxy-tamoxifen (TAM) (10 nM-1000 nM) alone or TAM plus 1.6 mg/mL JEKHT. Statistically significant *P*-values indicating that JEKHT sensitized LCC9 cells to increasing doses of TAM are shown. Mean ± s.e.m. are shown. Data were generated using three replicates of each exposure, and analyzed by *t*-test for each TAM dose separately.

To investigate if JEKHT also increases sensitivity of endocrine-resistant human breast cancer cells to TAM, we treated endocrine-resistant ER+ LCC9 breast cancer cells with 1.6 mg/mL JEKHT and increasing doses of 4-OHT *in vitro*. As seen in Fig. 2, TAM did not inhibit the growth of LCC9 cells. However, a significant and dose-dependent response was seen, starting with 125 nM dose of 4-OHT, when cells also were treated with JEKHT. Statistical significances are shown in Fig. 2.

### Effects on body weight and organ weight

#### TAM reduces body weight

**Figure 3:**
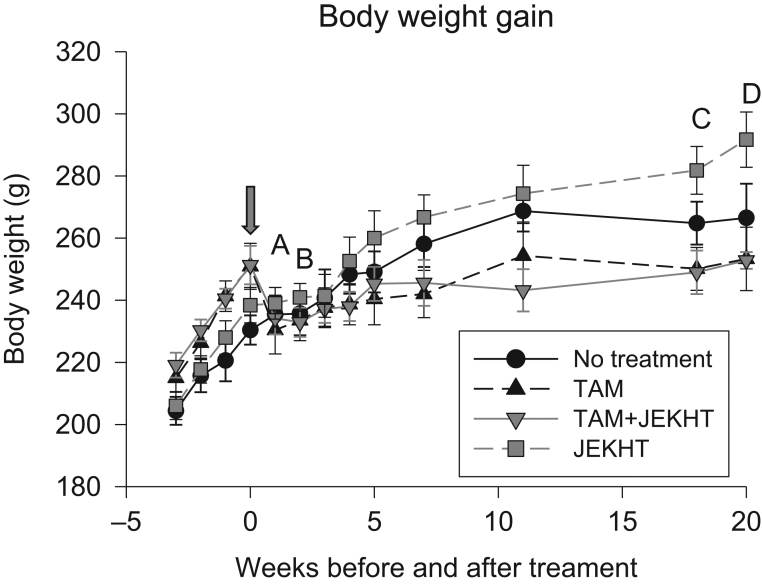
Effects on body weight development. The arrow indicates when tamoxifen (TAM) treatment started. TAM initially caused a significant weight loss that lasted for 2 weeks (A: week 0 vs 1 in TAM, *P* = 0.023 and in TAM + JEKHT, *P* = 0.025; B: week 0 vs 2 in TAM, *P* = 0.048 and in TAM + JEKHT, *P* = 0.036), after which TAM-treated animals started gaining weight. However, the weight gain was slower than in the JEKHT only group and a significant difference between TAM-treated and JEKHT only treated rats was seen on weeks 19 (C: JEKHT vs TAM, *P* = 0.009 and JEKHT vs TAM + JEKHT, *P* = 0.008) and 20 (D: JEKHT vs TAM, *P* = 0.008 and JEKHT vs TAM + JEKHT, *P* = 0.005) after starting the treatments. Data were analyzed using one-way ANOVA, followed by LSD test. Mean ± s.e.m. are shown, *n* = 7–10 rats per group.

When animals started receiving the treatments, those treated with either TAM or TAM + JEKHT exhibited a modest but significant reduction in their body weight (Fig. 3). The drop in body weights was seen after the 1st and 2nd week of treatments in both groups, subsequently rats started to gain weight again (statistical significances are shown in Fig. 3). Weight gain in these rats was slower than that in the rats that received JEKHT only. The difference in body weights between JEKHT only and the two TAM-treated groups reached statistical significance by weeks 19 and 20 of the treatments (Fig. 3). Body weights in the non-treated control group were slightly lower than in the JEKHT only group, and thus, were not different from the TAM-treated groups.

#### Organ weights were heavier in JEKHT group

At the end of tumor monitoring period, the weights of thymus, spleen and abdominal fat were significantly lower in the TAM and TAM + JEKHT-treated rats than in the JEKHT-treated rats (see Table 2 for statistical significances). These reductions likely reflect the lower body weights caused by TAM therapy.

**Table 2:** Effect of TAM, TAM + JEKHT or JEKHT treatments on weights of thymus, spleen and abdominal adipose depots in DMBA-exposed rats.

	TAM (*n* = 11)	TAM + JEKHT (*n* = 15–16)	JEKHT (*n* = 8)	One-way ANOVA
Thymus (mg)	17.9 ± 0.7*	16.2 ± 0.3**	19.1 ± 0.5	*P* = 0.002
Spleen (mg)	19.1 ± 0.9***	18.1 ± 1.0***	32.1 ± 3.5	*P* < 0.001
Abdominal fat (mg)	64.2 ± 6.2***	68.5 ± 4.4***	114.3 ± 7.3	*P* < 0.001

Means ± s.e.m. are shown.

Significantly different from JEKHT group: *P < 0.05; **P < 0.01; ***P < 0.001.

### Effects on the expression of ER-α, ER-β and PgR in TAM-treated mammary tumors

**Figure 4:**
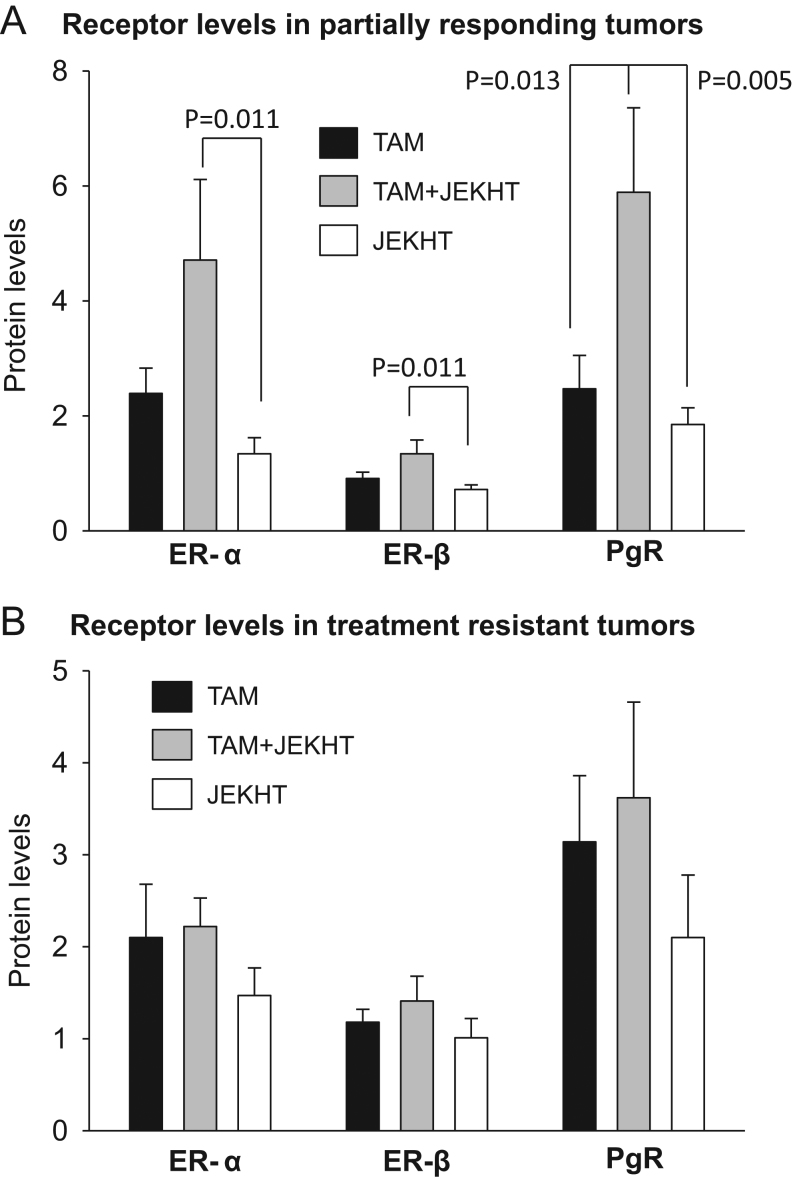
Effects on ERα, ERβ and PgR protein levels in mammary tumors. (A) Receptor levels (by Western blot) in partially responding and (B) treatment resistant mammary tumors in tamoxifen (TAM), JEKHT only, or TAM + JEKHT-treated rats. Compared with JEKHT only treated rats, partially responding tumors in rats treated with TAM + JEKHT exhibited increased ER-α, ER-β and PgR levels. Only PgR was significantly different between TAM only and TAM + JEKHT groups. Data were analyzed using one-way ANOVA, followed by LSD test. Statistically significant *P*-values are shown in the figure. Means ± s.e.m. of 5–6 PR and 5–9 *de novo* resistant tumors per group are shown.

We investigated the effects of JEKHT on ER and PgR in mammary tumors in the preclinical model. No differences in ER-α or ER-β levels in the partially responding or *de novo* TAM-resistant mammary tumors were seen between TAM and TAM + JEKHT-treated rats (Fig. 4A and B). However, PgR levels were significantly higher in partially responding tumors in the TAM + JEKHT group (Fig. 4A). Compared with the JEKHT only group, all three receptors were expressed at significantly higher levels in the rats treated with TAM + JEKHT in non-growing/partially responding tumors (see Fig. 4A for statistical significances), and no differences were seen between the TAM only and JEKHT only groups.

### Effects on cytokines and tumor immune microenvironment

#### Cytokine levels: circulation and spleen

Expression of IL-1b, IL-6, IL-10 and TNF-α is linked to increased breast carcinogenesis (Esquivel-Velazquez* et al.* 2015), although opposing findings also have been reported (Knupfer & Preiss 2007, Changkija & Konwar 2012). IFN-γ is secreted from many cells, including natural killer (NK) cells, naïve CD4 T cells and CD8+ cytotoxic T cells, and it activates antigen production to eliminate cancer cells (Bhat* et al.* 2017). TAM may increase, reduce or have no effects on these cytokines (Lindner* et al.* 1997, Barak* et al.* 1998, Behjati & Frank 2009). We found no evidence that these cytokine levels were altered in the circulation by giving JEKHT to TAM-treated rats, or by JEKHT alone, compared with TAM only treated rats (Table 3). In the spleen, IL-10 levels were increased in rats treated with JEKHT alone (see Table 3 for statistical differences). This increase may be caused by a rapid growth of mammary tumors in JEKHT only treated rats, rather than be a direct effect of JEKHT on IL-10.

**Table 3:** Effect of TAM, TAM + JEKHT or JEKHT on serum and spleen cytokine levels in DMBA exposed rats.

	TAM (*n* = 11)	TAM + JEKHT (*n* = 15–16)	JEKHT (*n* = 8)	One-way ANOVA
Serum cytokines				
IL-6 (pg/mL)	36.3 ± 1.2	36.5 ± 1.2	33.6 ± 0.8	ns
IFNγ (ng/mL)	64.8 ± 14.1	46.8 ± 8.9	34.9 ± 9.7	ns
Spleen cytokines				
IL-1b (pg/mL)	2069.4 ± 166.0	2129.8 ± 290.5	2542.3 ± 234.7	ns
IL-10 (pg/mL)	630.7 ± 50.3**	545.5 ± 31.8**	1016.8 ± 176.1	*P* = 0.005
TNFα (pg/mL)	81.0 ± 5.6	84.3 ± 4.8	98.9 ± 5.5	ns

Means ± s.e.m. are shown.

Significantly different from JEKHT group: **P < 0.01.

ns, not significant.

#### Cytokine levels: mammary tumors

**Figure 5:**
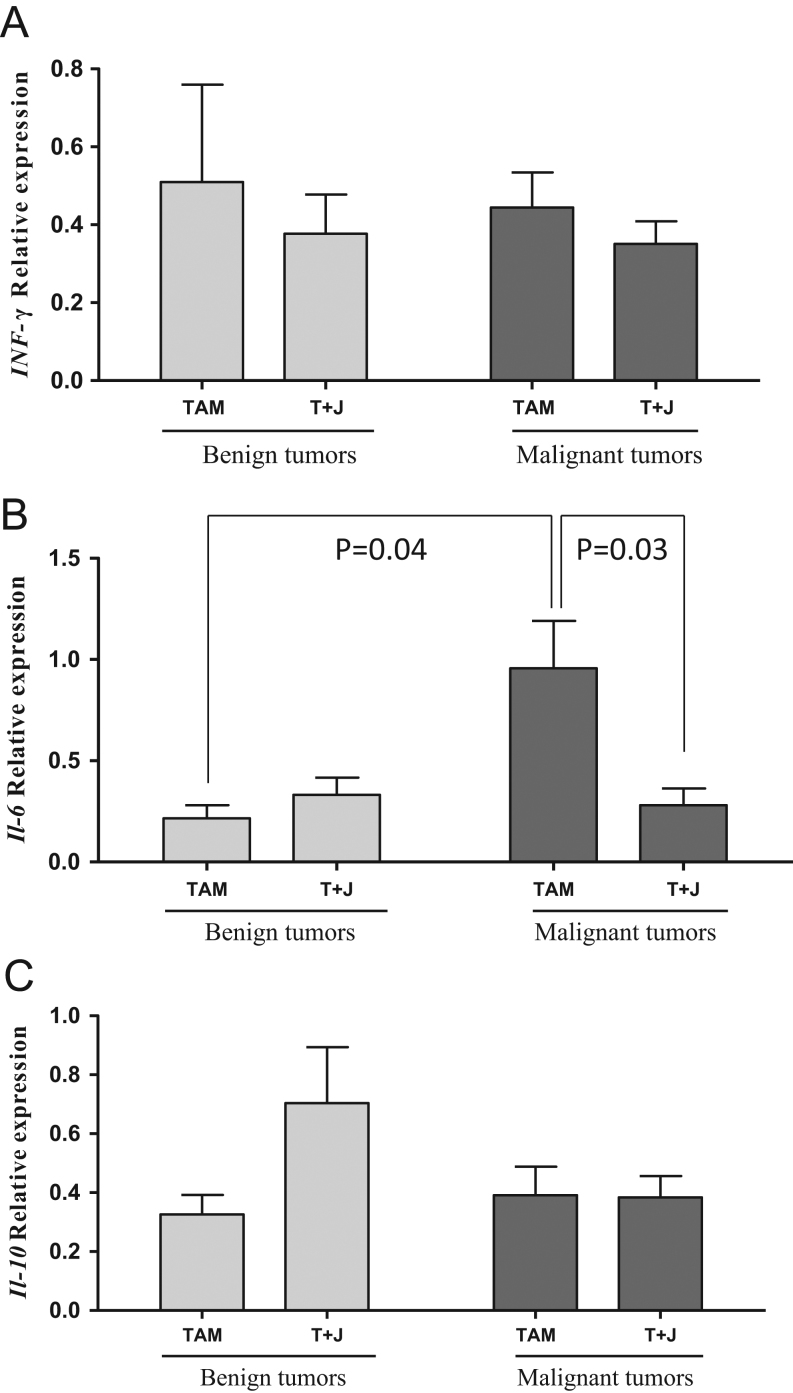
Effects on *Ifn-γ*, *Il-6* and *Il-10* in the mammary tumors. No significant differences in (A) *Ifn-*γ or (C) *Il-10* mRNA levels were seen in benign or malignant *de novo* resistant mammary tumors in rats receiving TAM or TAM+JEKHT treatment. (B) The expression of *Il-6* mRNA was significantly higher in malignant than benign TAM-treated tumors, and significantly reduced in malignant tumors in rats that were treated with TAM + JEKHT. Data were analyzed using two-way ANOVA, followed by Holm–Sidak test. Statistically significant *P*-values are shown in the figure. Means ± s.e.m. of 4–6 benign and 6–13 malignant *de novo* resistant tumors per group are shown.

Tumors from experiment 2 were used. Since over three-fourth of the tumors in animals treated with TAM and JEKHT exhibited complete response and were eliminated, we did not have enough tumor tissue available from partially responding tumors and only used resistant tumors to study mRNA levels of three key cytokines linked to immune cell functions: *Ifn-γ*, *Il-6* and *Il-10*. However, an unusually high number of benign tumors were seen among resistant tumors (34%), and thus, we assessed expression levels separately in benign and malignant mammary tumors. Treating rats with both TAM and JEKHT did not affect *Ifn-γ* or *Il-10* mRNA expression in mammary tumors (Fig. 5A and C). However, *Il-6* levels were significantly reduced by adding JEKHT to the treatment regimen in malignant tumors (*P* = 0.03) that also expressed significantly higher levels of *Il-6* than benign tumors (*P* = 0.04) (Fig. 5B).

### Effects on tumor immune microenvironment

#### CD8a protein levels

**Figure 6:**
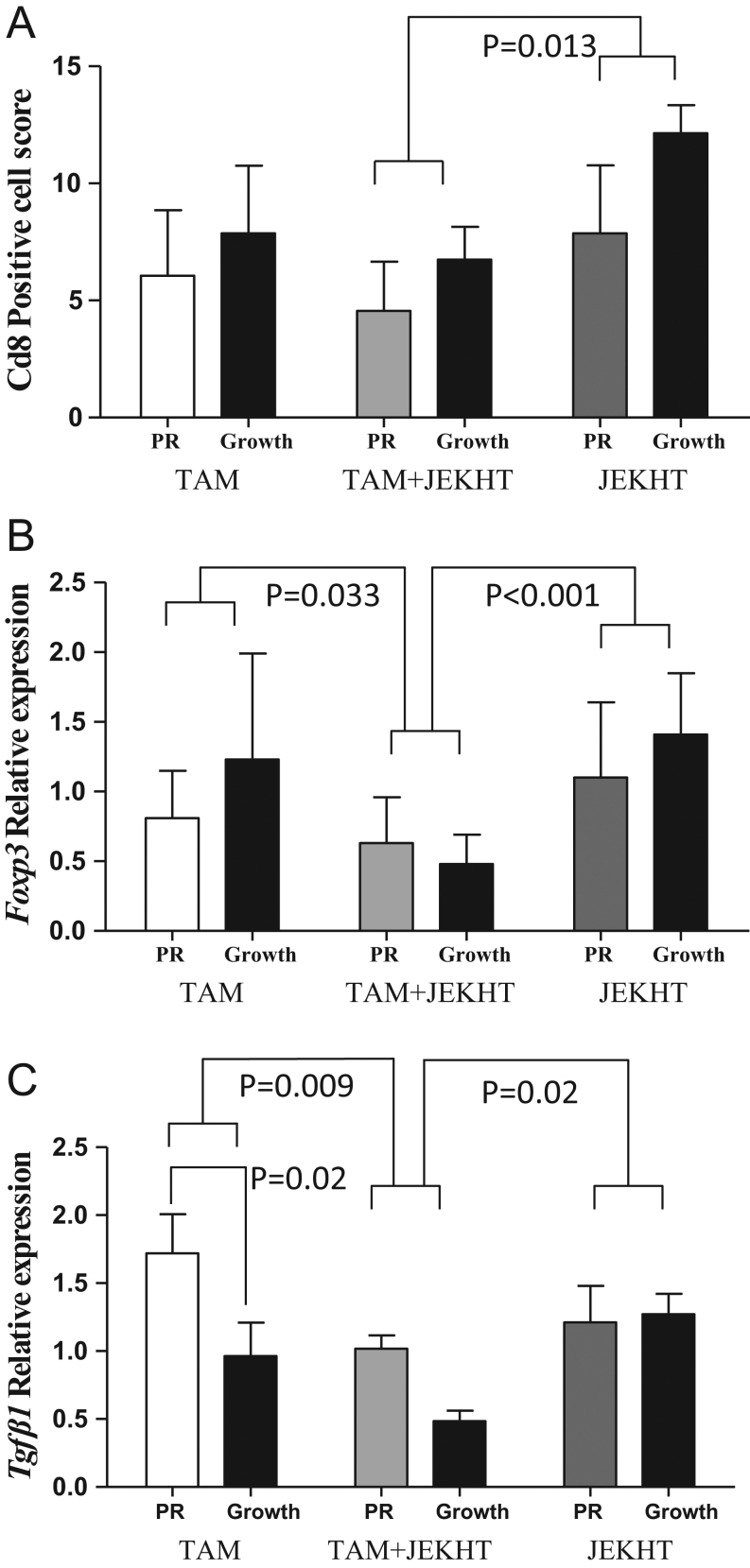
Effects on CD8+ T cells and expression of *Foxp3* and *Tgfβ1* in the mammary tumors. (A) JEKHT only treated rats exhibited more CD8+ lymphocytes in partially responding and *de novo* resistant mammary tumors than TAM + JEKHT-treated rats (*P* = 0.013). Assessment done by immunohistochemistry. (B) Rats treated with TAM + JEKHT exhibited lower *Foxp3* mRNA levels than TAM only (*P* = 0.033) or JEKHT only (*P* < 0.001) treated rats. (C) Rats treated with TAM + JEKHT exhibited significantly lower levels of *Tgfβ1*. Data were analyzed using two-way ANOVA, followed by Holm–Sidak test. Statistically significant *P*-values are shown in the figure. Means ± s.e.m. of 3–4 PR and 4 *de novo* resistant tumors per group are shown.

Tumors from experiment 1 were used to study the effector and regulatory T cell markers. In this study, all tumors used were malignant. Protein levels of the T effector cell marker CD8a, assessed by IHC, were not different in mammary tumors between TAM and TAM + JEKHT-treated rats (Fig. 6A). However, rats that only received JEKHT exhibited significantly higher tumor infiltration of CD8a+ T cells than rats receiving both TAM and JEKHT (*P* = 0.013) (Fig. 6A). No differences between partially responding and resistant tumors in any of the treatment groups were seen.

#### *Foxp3* mRNA

Adding JEKHT to the drinking water of TAM-treated rats reduced *Foxp3* levels (Fig. 6B). *Foxp3* mRNA expression was significantly lower in the TAM + JEKHT-treated rats than in the TAM only (*P* = 0.033) or JEKHT only (*P* < 0.001) treated rats (Fig. 6B). No differences between partially responding and resistant tumors in any of the treatment groups were seen.

#### *Tgfβ1* mRNA

Expression of *Tgfβ1* was significantly reduced by adding JEKHT to TAM treatment, when compared with TAM only (*P* = 0.009) or JEKHT only groups (*P* = 0.02) (Fig. 6C). In addition, TAM-resistant tumors exhibited lower levels of *Tgfβ1* than responsive tumors (*P* = 0.02) (Fig. 6C).

### Effects on the endometrium

#### JEKHT prevents TAM-induced premalignant endometrial lesions

Similar to humans, TAM induces hyperplasia in the endometrium in animal models (Sourla* et al.* 1997). Oral gavage of DMBA is not reported to induce endometrial cancer, but endometrial tissues appeared slightly or moderately inflamed in all DMBA-exposed animals. The JEKHT only group exhibited significantly more moderate inflammation (57%) than the TAM only group (18%) or TAM + JEKHT group (10%) (*P* < 0.001). These animals (71%) and rats treated with TAM + JEKHT (90%) exhibited significantly more benign hyperplasia than the TAM only treated rats (54%) (*P* < 0.001). However, almost 20% of rats treated with TAM only had developed pre-neoplastic changes, while none were seen in JEKHT + TAM or JEKHT only groups. Pre-neoplastic foci were characterized by the presence of hyperplasia with atypia.

#### No changes in the expression of ER-α, ER-β or PgR in TAM-treated endometrial tissue

TAM acts as an ER agonist in the uterus but an earlier study found no changes in the expression of hormone receptors in the rat uterus by TAM therapy (Bayram* et al.* 2005). Consistent with these data, in the present study no changes were seen in ER-α, ER-β or PgR levels in the endometrial tissues among the rats treated with TAM, JEKHT or both (data not shown).

## Discussion

JEKHT, a preparation composed of 12 medical herbs, has been used for centuries in Asia for multiple health problems, including to treat inflammation and boost immune responses (Kim *et al.* 2004, 2015). Similar to other herbs, or herb combinations, JEKHT has been reported to inhibit the growth of cancer cells. Specifically, JEKHT inhibits human HT1080 fibrosarcoma cells, PC-3 prostate cancer cells and AGS gastric carcinoma cells at the doses of 0.5 and 1.0 mg/mL *in vitro* (Kim* et al.* 2015). In animal models, 120 mg/kg body weight JEKHT inhibited the growth of HT1080 fibrosarcoma cells in mice (Kim* et al.* 2015), and 200 or 400 mg/kg body weight JEKHT prevented the development of benign prostatic hyperplasia in rats (Shin* et al.* 2012). In the latter study, JEKHT induced apoptosis and downregulated several genes linked to cell proliferation (Shin* et al.* 2012).

Our study is the first to investigate the interactions between antiestrogen therapy and herbal mixture in ER+ breast cancer *in vivo* using a model that allows studying both primary response and risk of recurrence in fully immunocompetent animals (Hilakivi-Clarke* et al.* 2016). By using this model, we have previously shown that dietary intake of an isoflavone genistein reduced both *de novo* and acquired TAM resistance (Zhang* et al.* 2017). However, genistein had to be consumed before tumors had developed to protect against breast cancer recurrence in TAM-treated rats; consumption that started for the first time during TAM therapy increased the risk of recurrence (Zhang* et al.* 2017). Here, we studied the interaction between TAM and JEKHT when both were administered simultaneously. Starting JEKHT for the first time with TAM increased sensitivity to TAM. Thus, JEKHT could be given during TAM treatment and prevent the local recurrence of ER+ breast cancers. Importantly, we observed a significant increase in complete responses in two separate experiments done using two different batches of JEKHT, suggesting that differences in the percent of some herbs in the mixture did not appear to have affected its efficacy. As there are likely to be similar differences in JEKHT mixtures prepared by different manufactures, our results suggest that these differences do not impact the ability of JEKHT to promote responsiveness to tamoxifen therapy. If JEKHT was given as a monotherapy, it increased the proportion of tumors that stopped growing. However, most tumors in JEKHT only treated rats continued to grow. In an earlier study, plumbagin, a naturally occurring yellow pigment in the roots of a flowering medicinal plant *Plumbago indica*, enhanced TAM sensitivity of MCF-7 and T47D human breast cancer cells (Kawiak* et al.* 2017) and sensitized antiestrogen resistant human breast cancer cells to TAM *in vitro* (Sakunrangsit* et al.* 2016). We found that endocrine-resistant LCC9 human breast cancer cells began to respond to TAM if co-treated with JEKHT. These results suggest that JEKHT might reduce the development of endocrine resistance in ER+ breast cancer patients.

When assessing changes in tumor growth in cancer cells that are treated both with a standard Western and alternative therapy, it is critical to determine if the alternative treatment adversely affects the metabolism of the traditional treatments. Treating male rats with a single dose of TAM (50 mg/kg) and JEKHT (100 mg/kg) did not influence TAM pharmacokinetics (Kwak* et al.* 2016). The findings reported in this study are thus unlikely to be caused by changes in TAM metabolism. We did not observe any adverse health effects of JEKHT treatment either body weight gain and organ weights of animals that received TAM + JEKHT were similar to those of animals treated with TAM only. JEKHT only increased body weight gain as well as weight of specific organs and tissues (thymus, spleen and abdominal fat), compared with animals receiving no treatment or those treated with TAM or TAM + JEKHT. Whether JEKHT may benefit cancer patients suffering from cachexia-induced weight loss is not known, but several other herbs are used by traditional Asian medicine practitioners for that purpose (Ming-Hua* et al.* 2016).

To identify the potential mechanisms leading JEKHT to increase sensitivity to TAM, we first measured protein levels of ERα, ERβ and PgR in the mammary tumors. All three receptors were upregulated in partially responding tumors in rats treated with TAM + JEKHT, compared with the JEKHT only group, but only PgR was significantly higher in the tumors of TAM + JEKHT group than TAM only group. Since in premenopausal breast cancer patients receiving adjuvant TAM therapy, high pre-treatment PgR levels were predictive of longer recurrence-free survival (Stendahl* et al.* 2006, Chapman* et al.* 2013), upregulation of PgR in the TAM + JEKHT group may be causally related to their increased TAM sensitivity and reduced risk of recurrence.

Another possible mechanism explaining increased sensitivity to TAM is a change in cytokines and tumor immune parameters by JEKHT. KIOM-C, another herbal mixture that contains some of the same herbs as JEKHT (*Radix Glycyrrhizae* and *Radix Angelicae Gigantis*) and has anti-metastatic activity in cancer cells, increased serum IFN-γ levels in nude mice (Kim* et al.* 2014). We found no significant changes in serum IL-6 or IFN-γ levels among the rats treated with TAM, JEKHT or their combination. In the spleen, the contents of IL-1β and TNF-α were not altered, but IL-10 levels were increased in the JEKHT only treated group, compared with the other groups. IL-10 is an anti-inflammatory cytokine released from multiple types of immune cells (Saraiva & O’Garra 2010). While IL-10 stimulates immunosuppressive FOXP3 expressing T regulatory (Treg) cells (Murai* et al.* 2009), its role in breast cancer remains unclear, as it has been reported to both promote and inhibit breast cancer growth (Changkija & Konwar 2012).

We also assessed the expression of *Ifn-γ*, *Il-6* and *Il-10* in mammary tumors. In malignant mammary tumors, *Il-6* was significantly downregulated by treatment with TAM + JEKHT, compared with the TAM only group. IL-6 is an inflammatory cytokine, but it also assists in inhibiting immunosuppressive Foxp3/Treg cells (La 2008). Similar to IL-10, IL-6 may act to promote or suppress breast cancer (Knupfer & Preiss 2007). However, since IL-6 also stimulates mammary cancer stem cells, probably as a consequence of a natural inflammatory repair program to activate stem cells to replace cells eliminated by cytotoxic CD8+ T cells (Sansone* et al.* 2007), its suppression in mammary tumors by JEKHT in TAM-treated animals may be involved in improving TAM response.

Little is known about the role of the immune system in endocrine resistance. TAM is reported to have multiple effects on immunity. For example, TAM shifts CD4+ T cells from T helper 1 (Th1) to Th2 immunity (Behjati & Frank 2009), and thus, can promote pro-tumor immunosuppression. TAM also downregulates cytotoxic CD8+ T effector cells and upregulates Tregs in human breast cancer cells (Joffroy* et al.* 2010). However, impaired anti-tumor immunity by TAM may occur mostly in those women with an elevated Th2 immunity evident prior to treatment (Li* et al.* 2015). Although upregulation of Foxp3/Treg cells is predictive of poor outcome in all receptor types of breast cancer (Liu* et al.* 2014, Miyashita* et al.* 2014), CD8+ T cells are not linked to prognosis in ER+ disease (Dushyanthen* et al.* 2015). In our study, no differences in CD8+ T cell levels were seen between TAM only and TAM+JEKHT-treated rats. However, JEKHT upregulated CD8+ T cells, compared with TAM + JEKHT-treated rats, suggesting a higher level of anti-tumor immune responses.

Rats treated with TAM + JEKHT exhibited significantly lower levels of *Foxp3* mRNA in mammary tumors than TAM only or JEKHT only groups, which is indicative of a lower level of immunosuppression. *Tgfβ1* mRNA expression also was downregulated in the mammary tumors of TAM + JEKHT-treated rats, compared with the other two groups of rats. TGFβ1 activates Treg cells (Buck & Knabbe 2006). Thus, our findings imply that JEKHT may prevent the immunosuppressive effects of TAM and reduce some of the adverse effects of TAM on the immune system, promoting anti-tumor immunity. The higher rates of responses and lower risk of recurrence observed in the combination group is likely related to tumor immune responses.

Herbal preparations, such as Jia-Wei-Xiao-Yao-San or Shu-Jing-Huo-Xue-Tang (Tsai* et al.* 2014, Hu* et al.* 2015) or Ginseng (Hsu* et al.* 2015), can prevent TAM’s adverse effects on the endometrium in breast cancer patients. While our animal model was not designed to measure their effects on endometrial cancer, we were able to determine TAM’s ability to induce premalignant lesions in the endometrium – hyperplasia with atypia (Burke* et al.* 1996). Nineteen percent of TAM only treated rats exhibited these premalignant lesions, while none were seen in rats that received both TAM and JEKHT or JEKHT only. Thus, in addition to reducing the risk of developing TAM resistance and risk of recurrence in our experimental animal model, adding JEKHT to the treatment regimen may also protect the uterus against the adverse effects of TAM.

## Supplementary Material

Supplementary Table 1

## Declaration of interest

The authors declare that there is no conflict of interest that could be perceived as prejudicing the impartiality of the research reported.

## Funding

Study funded by The Ministry of Health & Welfare (MOHW), Republic of Korea (Grant Number: 090-091-3000-3038-301-320-01) Comprehensive and Integrative Medicine R&D project through Comprehensive and Integrative Medicine Institute (CIMI) to K FitzGerald, U01-CA184902 to R Clarke, R01-CA164384 to L Hilakivi-Clarke and P30-CA51008 Cancer Center Support grant to Dr L Weiner.

## Ethics approval

All animal procedures were approved by the Georgetown University Animal Care and Use Committee (GUACUC).

## Author contribution statement

L H-C is the principal investigator of the study and study supervisor. She is responsible of all aspects of the study. L H-C, F D O A, R C and K F conceptualized and designed the study and wrote and reviewed the manuscript. W Y, F D O A, R C and L H-C developed the methodology for studying ER+ breast cancer using syngeneic preclinical model. F D O A, W Y, X Z were responsible for performing all aspects of the animal study, including TAM treatment, JEKHT administration via drinking water, monitoring tumorigenesis and collection of tissues and their processing. R H performed studies involving LCC9 human breast cancer cell lines. F D O A, W Y and X Z performed all molecular biology studies. F D O A, W Y and L H-C performed statistical analysis.
