# Supplementary material for: Effects of Jaeumkanghwa-tang on tamoxifen responsiveness in preclinical ER+ breast cancer model
Source: Endocr Relat Cancer. 2019 Jan 14;26(3):339–53. doi: 10.1530/ERC-18-0393 (PMC6365679; doi:10.1530/ERC-18-0393)
Supplement: Supplementary Table 1 [file supplementary_table_1.pdf]

Supplemental Table 1. Primer sequences

**Gene ID**	**Sequence**
Foxp3 _forward	5’-TGCCACCTGGGATCAATGTG -3’
Foxp3 _reverse	5’-CGTGGGAAGGTGCAGAGTAGAGC -3’
Il-6 _forward	5’- TTGCCTTCTTGGGACTGATG-3’
Il-6 _reverse	5’-GTGGTATCCTCTGTGAAGTCTC -3’
Il-10 _forward	5’-AAAGCAAGGCAGTGGAGCAG -3’
Il-10 _reverse	5’-TCAAACTCATTCATGGCCTTGT -3’
IFNγ _ forward	5’-CCAAGGCACACTCATTGAAAG -3’
IFNγ _ reverse	5’- TCTGGCTCTCAAGTATTTTCGTG-3’
Tgfβ1 _ forward	5’- CCTGAGTGGCTGTCTTTTGA-3’
Tgfβ1 _ reverse	5’-CGTGGAGTACATTATCTTTGCTG -3’
Hprt1 _ forward	5’-GCCCTTGACTATAATGAGCACT -3’
Hprt1 _ reverse	5’-CCGCTGTCTTTTAGGCTTTG -3’
